# Frontotemporal dementia: a neurophysiological study

**DOI:** 10.18632/aging.101604

**Published:** 2018-10-18

**Authors:** Flavio Di Stasio, Antonio Suppa, Alfredo Berardelli

**Affiliations:** 1 IRCCS Neuromed Institute, Pozzilli (IS), Rome, Italy; 2 Department of Human Neurosciences, Sapienza University of Rome, Rome, Italy

**Keywords:** frontotemporal dementia, parkinsonism, cortical plasticity, primary motor cortex, transcranial magnetic stimulation, theta-burst stimulation

Frontotemporal dementia (FTD) is a neurodegenerative disorder clinically characterised by progressively worsening deficits in behaviour, personality, executive function and language [1]. Clinical variants of FTD include the behavioural variant (bv-FTD), which is mainly characterized by disinhibition, compulsion and personality changes, and the primary progressive aphasia variant (PPA-FTD), which is characterized by deficits in linguistic skills. However, in addition to the well-known changes in behaviour, cognition and language, up to 15% of FTD patients may have signs and symptoms of upper and/or lower motor neuron disease, such as hyperreflexia, spasticity, muscle weakness, atrophy and fasciculation [1], which are indicative of the FTD-amyotrophic lateral sclerosis (FTD-ALS) complex. Moreover, approximately 20% of FTD patients may exhibit motor symptoms including parkinsonism [1]. The possible coexistence of cognitive, behavioural and motor symptoms makes FTD a heterogeneous, complex disorder that often overlaps other conditions such as progressive supranuclear palsy (PSP) and corticobasal syndrome (CBS). Although histopathological studies have demonstrated that FTD, PSP and CBS largely share the same pathological hallmark, i.e. brain deposition of tau-protein, the pathophysiological mechanisms leading to these neurodegenerative conditions remain unclear. Moreover, since parkinsonism is known to occur both in FTD and in other tauopathies such as PSP and CBS [2], any neurophysiological differences and similarities between FTD and other tau-related atypical parkinsonisms need to be investigated to gain a better understanding of the specific pathophysiological mechanisms underlying these neurodegenerative disorders [3].

The pathophysiological mechanisms responsible for parkinsonism in FTD were specifically investigated in a transcranial magnetic stimulation (TMS) study by Di Stasio et al. (2018) [4] that was recently published in Neurobiology of Aging. The FTD patients in that study underwent the "Theta Burst Stimulation" technique [5,6], a non-invasive brain stimulation protocol that elicits long-term potentiation (LTP)-like plasticity and long-term depression (LTD)-like plasticity in the primary motor cortex (M1) depending on what parameters are used. Intermittent TBS (iTBS) increases the amplitude of motor-evoked potentials (MEPs) for about 60 minutes, whereas continuous TBS (cTBS) reduces MEPs owing to LTP- and LTD-like plasticity, respectively [5,6]. Investigating M1 plasticity in patients with motor symptoms is an issue of considerable scientific relevance since LTP/LTD are physiological mechanisms that have been widely acknowledged to underlie motor execution and learning. In order to be able to discriminate the pathophysiological mechanisms related to FTD *per se* from those that may be responsible for parkinsonian symptoms, Di Stasio et al. (2018) [4] examined and compared motor responses to iTBS and cTBS in patients with and without parkinsonism. A crucial point to bear in mind is that patients with clinical and/or instrumental upper or lower motor neuron involvement were excluded. The main finding of the study was that FTD patients with parkinsonism respond abnormally to TBS, whereas those without parkinsonism do not. The results obtained in FTD patients with parkinsonism are likely due to neurodegenerative processes in the frontal regions, including M1 and the cortico-basal ganglia-thalamo-cortical motor loops, which is in keeping with results from neuroimaging studies [7]. By contrast, the normal LTP/LTD-like plasticity observed in FTD patients without parkinsonism suggests that these patients do not have an involvement of the cortico-basal ganglia-thalamo-cortical loop (including M1). Hence, in FTD patients, parkinsonian symptoms such as rigidity and bradykinesia are presumably related to abnormal LTP/LTD-like plasticity in M1.

Several research issues in the pathophysiology of FTD have yet to be fully investigated and clarified. The neurophysiological abnormalities reported in the FTD patients studied by Di Stasio et al. (2018) [4] are not disease-speciﬁc. Responses to iTBS and cTBS are also known to be altered in other neurodegenerative disorders: reduced in patients with Parkinson’s disease (PD) and multiple system atrophy (MSA) but increased in patients with atypical parkinsonisms such as PSP and specific variants of CBS [6]. Hence, any differences and similarities in responses to TBS between FTD and other neurodegenerative disorders characterized by atypical parkinsonism should be interpreted bearing in mind that these disorders arise from complex and heterogeneous patterns of neurodegenerative processes not only in the basal ganglia but also in various cortical motor and non-motor areas. A further important question concerns the specific pathophysiological link between neurophysiological abnormalities and the clinical signs and symptoms in patients with FTD. Since the parkinsonian features manifested by patients with FTD include bradykinesia and rigidity though not tremor, we suggest that bradykinesia and rigidity result from a disorder of LTP/LTD-like plasticity in M1 owing to abnormal motor inputs driven by the cortico-basal ganglia-thalamo-cortical loops, whereas tremor does not [4]. Studies that combine advanced neurophysiological tools and structural and functional neuroimaging techniques may help to identify specific pathophysiological changes in patients with various subtypes of FTD and thus gain a better insight into the pathophysiology of specific motor and non-motor symptoms in this disorder.
